# Understanding Mental Health Challenges in Cardiovascular Care

**DOI:** 10.7759/cureus.54402

**Published:** 2024-02-18

**Authors:** Pawel Borkowski, Natalia Borkowska

**Affiliations:** 1 Internal Medicine, Albert Einstein College of Medicine, Jacobi Medical Center, New York, USA; 2 Pediatrics, SPZOZ Krotoszyn, Krotoszyn, POL

**Keywords:** holistic medicine, integrated care model, heart-brain connection, mental well-being, cardiovascular health

## Abstract

There is a profound link between cardiovascular health and mental well-being. This narrative review shows that heart and mental health are not isolated domains but deeply interconnected, influencing each other. It describes how cardiovascular diseases (CVDs) can cause mental health issues such as stress, anxiety, and depression. It also explains how these mental conditions can, in turn, worsen or raise the risk of CVDs. In addition, it emphasizes the necessity of a holistic approach to healthcare that integrates the treatment of physical symptoms of CVDs with interventions aimed at addressing mental health issues. This approach advocates for comprehensive care strategies that include psychotherapy, pharmacological treatments, lifestyle modifications, and digital health technologies. It also highlights the significant role of family and social support in recovery and discusses barriers to integrating mental health care in cardiovascular treatment. The article argues for a paradigm shift in healthcare towards more inclusive and integrated care models. The authors hope to foster a healthcare environment that prioritizes holistic care by increasing awareness about the connection between heart and mind. The call to action includes policy changes and healthcare system reforms aimed at facilitating the integration of mental health services into cardiovascular care, ultimately leading to improved outcomes for patients with CVDs and associated mental health issues.

## Introduction and background

Cardiovascular diseases (CVDs) refer to conditions that affect the heart and blood vessels worldwide [1]. These include coronary artery disease (CAD), heart failure (HF), irregular rhythms, rheumatic heart disease, congenital heart disease, cerebrovascular disease, rheumatic heart disease, peripheral arterial disease, deep vein thrombosis, and pulmonary embolism. These diseases share risk factors (e.g. hypertension, hyperlipidemia, diabetes, smoking, sedentary lifestyle) and can lead to significant complications (e.g. organ damage and disability). According to data from the World Health Organization (WHO), CVDs are the leading cause of death worldwide [1]. In 2019, about 17.9 million people died from CVDs, making up 32% of all reported deaths [1]. Myocardial infarction (MI) and stroke accounted for around 85% of these cases. The WHO also estimates that in the United States (US), CVDs continue to be the predominant cause of mortality, with a reported nearly 1 million deaths in 2020. People with CVDs often experience a decreased Quality of Life (QOL) [2]. Research indicates that CVD patients with higher comorbidity levels often experience more depressive symptoms, physical limitations, and a decline in overall health status [3,4]. In addition, the financial implications of CVDs between 2018 and 2019 were significant as they resulted in an expenditure of $407.3 billion ($251.4 billion in direct healthcare expenses and $155.9 billion attributed to lost productivity and mortality) [5].

Living with CVDs goes beyond just the physical symptoms. It presents challenges that deeply affect a patient’s life [6]. This causes a lot of psychological challenges to the patients. For example, diagnosis with CVDs can lead to vigilance, uncertainty about one’s health, and a sense of vulnerability. Individuals in this situation may constantly worry about complications leading to stress and anxiety that can impact all aspects of their lives [7]. This can disrupt sleep, impair concentration, and decrease the ability to enjoy leisure activities. Moreover, the limitations imposed by the condition, such as reduced physical capabilities and the need for treatment, can lead to feelings of frustration and helplessness, which may eventually lead to depression [8].

The objective of this narrative review is to comprehensively examine the connection between mental health and CVDs, highlighting the bidirectional influence they have on each other and their impact on individuals. It also outlines effective management strategies encompassing pharmacological treatments, psychotherapy, lifestyle modifications, and digital health technologies. It also advocates for policy changes and healthcare system reforms to support integrating mental health services into cardiovascular care for improved patient outcomes.

## Review

Understanding the link between heart health and mental well-being

One of the most immediate reactions to CVDs is stress, which is a natural reaction to receiving a diagnosis. The nature of the long-term illness and its management cause chronic stress that can be detrimental [9]. Continuous stress and worries cause anxiety, which can disrupt daily routines, sleep patterns, and social interactions. For instance, Todaro et al. conducted research on 150 patients diagnosed with coronary heart disease to determine the prevalence of anxiety disorders [10]. They discovered that 45.3% of these patients (68 individuals) had experienced an anxiety disorder at some point in their lives. Social phobia and generalized anxiety disorder emerged as the most commonly observed, each with a lifetime prevalence rate of 26%. In comparison, lower prevalence rates were noted for panic disorder (5.3%), agoraphobia (4.7%), posttraumatic stress disorder (1.5%), and obsessive-compulsive disorder (0.7%). Moreover, some patients with CVDs may experience depression, which arises from a sense of loss - loss of health, loss of independence, and loss of the ability to participate in previously enjoyed activities. Colquhoun et al. found that approximately 15% of patients have major depressive disorder following MI or coronary artery bypass grafting [11]. This can profoundly impact the QOL, leading to social withdrawal. In addition, individuals suffering from depression have a higher likelihood of CVDs and mortality rates compared to the general population. In a comprehensive study with 145,862 participants, Rajan et al. determined that depression was linked with new cases of CVDs (HR: 1.14, 95%, CI: 1.05-1.24) and higher all-cause mortality (HR: 1.17, 95%, CI: 1.11-1.25) [12]. Moreover, anxiety disorders and depression often lead to reduced physical activity, increased smoking and alcohol intake, and drug abuse, which can further deteriorate health [13-16]. Living with CVDs can also affect relationships and employment. Patients may find it challenging to express their needs and emotions, leading to miscommunication and strained relationships. Ford et al.'s study showing how social connections benefit CVDs prevention supports this evidence [17]. In general, the psychological impacts of CVDs underscore the importance of care that addresses not just physical well-being, but also mental and emotional health.

Several biological mechanisms are suggested to play a role in the interplay between mental disorders and CVDs. A key factor in this link is the activation of the hypothalamic-pituitary-adrenal (HPA) axis from persistent stress and anxiety, leading to elevated cortisol levels [18]. High cortisol, along with altered cortisol stress reactivity, are commonly observed in disorders such as depression and anxiety disorders [18]. Persistently high cortisol levels cause dysfunction in endothelial regulation, disbalance of pro- and anti-inflammatory interleukins, and the recruitment of circulating monocytes to the arterial wall [19]. All these mechanisms promote the formation of atherosclerotic plaques. It also disrupts glucose regulation, which may progress to hyperinsulinemia and insulin resistance, which can further progress into diabetes [20]. In addition, cortisol itself also induces psychiatric disturbances, thereby perpetuating a vicious cycle [21]. Furthermore, chronic stress and anxiety disorders contribute to sustained activation of the sympathetic nervous system. This prolonged hyperadrenergic state accelerates vascular inflammation and oxidative stress. As a result, hypertension and, eventually, atherosclerosis develop, which cumulatively enhance the progression of CVDs [22]. Additionally, the research shows that elevated levels of inflammatory biomarkers (e.g. interleukin (IL)-1, IL-6, and C-reactive protein) are associated with both CVDs and depression [23,24]. This emphasizes the importance of inflammation in the pathophysiology of mental disorders and CVDs.

Understanding the link between CVDs and mental well-being involves recognizing the bi-directional relationship between cardiovascular and mental health [25]. This concept emphasizes that physical health issues can worsen mental health conditions and vice versa. Early recognition and treatment of mental health issues are vital in preventing heart-related complications while managing heart health is crucial for mental well-being. In addition, this interplay highlights the necessity for a holistic approach in healthcare, addressing both mental, physical, and social aspects to promote overall well-being.

Strategies for managing mental health in cardiovascular patients

It is essential to take an integrated approach that addresses both the physical and mental health needs of patients. This approach should include multiple interventions. Among various psychotherapeutic approaches, cognitive behavioral therapy (CBT) has received the most attention and research in the CVD patient group. For example, it has proven effective for CAD patients [26,27]. Namely, in a systematic review and meta-analysis conducted by Li et al. (4991 subjects with CAD), it was demonstrated that CBT can effectively reduce depression symptoms by -2.00 (95%, CI: -2.83 to -1.16, p < 0.001), anxiety symptoms by -2.07 (95%, CI: -3.39 to -0.75, p = 0.002), and stress symptoms by -3.33 (95%, CI: -4.23 to -2.44, p < 0.001) [26]. Additionally, these interventions can decrease body mass index by -0.47 (95%, CI: -0.81 to -0.13, p = 0.006). Pharmacological treatments can complement psychotherapeutic interventions. Tricyclic antidepressants (TCAs) are often not recommended for patients with CVDs due to their association with adverse effects such as orthostatic hypotension, tachycardia, and an increased risk of arrhythmias, with cardiovascular complications reported in both individuals with pre-existing CVDs and those without any history of cardiac conditions [28,29]. Conversely, selective serotonin reuptake inhibitors (SSRIs) are considered safe for managing psychiatric disorders in patients with CVDs [28]. However, it is important to note that SSRIs may also present side effects, including orthostatic hypotension, mild bradycardia, and QT interval prolongation, although these are generally less severe compared to those associated with TCAs [30,31]. Beyond their application in treating depression, SSRIs have been shown to potentially mitigate cardiovascular risks. For instance, a case-control study by Sauer et al., involving 5,336 patients, found that fluoxetine, sertraline, or paroxetine use was linked to a lower risk of MI, with an odds ratio of 0.59 (95%, CI: 0.39-0.91, p = 0.02), likely due to their ability to reduce platelet activation by limiting serotonin storage [32]. Lifestyle changes (healthy diet, regular exercise, avoiding toxic habits) also significantly improve mental health [33]. In addition, cardiac rehabilitation programs also play a role in the integrated care approach. These programs, traditionally focused on physical recovery, have shown significant psychological benefits [34]. They help reduce depression and anxiety among CVD patients by providing structured exercise, education about heart-healthy living, and emotional support. In conclusion, a comprehensive treatment plan for cardiovascular patients ideally encompasses psychotherapy, pharmacotherapy, lifestyle modifications, and cardiac rehabilitation. This holistic approach is best achieved through care models that utilize multidisciplinary teams.

The impact of digital health technologies on patient care

The advent of digital technologies has significantly transformed patient care, particularly in the management of CVDs and mental health. The WHO defines telehealth as “the delivery of health care services, where distance is a critical factor, by all health care professionals using information and communication technologies for the exchange of valid information for diagnosis, treatment, and prevention of disease and injuries, research and evaluation, and for the continuing education of health care professionals, all in the interests of advancing the health of individuals and their communities” [35]. Telehealth is commonly used across the US, particularly amplified during the COVID-19 pandemic [36]. It has revolutionized healthcare by offering patients consistent access to healthcare professionals without the need for physical travel, which is especially beneficial for those with mobility issues or living in remote areas. For example, telehealth has been effectively utilized in managing various CVDs, including arrhythmias, HF, hypertension, and CAD [37]. Telehealth enhances clinical outcomes and positively impacts patient involvement and satisfaction, enabling patients to take a more active role in managing their health conditions. Additionally, mobile health apps have emerged as tools for self-management and education [38]. These apps can track vital signs, medication adherence, and symptoms, providing patients with real-time feedback and personalized health advice. They also serve as platforms for mental health support, offering resources such as stress management techniques and cognitive behavioral therapy. Together, the digital health technologies have enhanced the quality and accessibility of healthcare. They encourage patients to actively manage their health and foster a proactive and preventative approach to health.

Barriers to digital health include a lack of technology and reimbursement challenges [37]. Around a quarter of adults in America lack access to broadband internet [39]. Improvement in access, especially in rural areas, is crucial, and public policy changes are needed to address this digital divide. This could involve government investment in digital infrastructure, particularly in underserved areas, and incentivizing private companies to expand high-speed internet coverage. To address telehealth reimbursement challenges, two strategies are essential - first, the implementation of national reimbursement policies would ensure fair coverage across states; second, creating a clear billing framework would guarantee appropriate compensation for healthcare providers. These measures would simplify the reimbursement process, benefiting both providers and patients. Lastly, the absence of standardized methods for assessing telehealth quality is a significant problem. Effective telehealth quality assessment involves patient satisfaction surveys, health outcome tracking, response time measurement, and adherence to privacy standards.

The role of family and social support in recovery

Family, friends, and community support are essential in CVD patients' recovery and overall well-being. For example, Okkonen et al.'s study of 279 coronary artery bypass surgery patients revealed that lower pre-surgery family support was linked to increased depression and anxiety compared to those with higher levels of support [40]. The support system provides more than just emotional comfort. It influences the patient's psychological health, impacting their recovery and QOL. Family members and friends offer essential daily support, from assisting with medication management and doctor's appointments to providing encouragement and understanding during challenging times. Their presence can alleviate feelings of isolation and loneliness, which are common among patients dealing with long-term illnesses. Social support plays a role in the positive health outcomes of individuals with CVDs by fostering a sense of belonging and offering opportunities for social interaction, yet effective strategies to boost social support in this group remain largely unexplored [41]. In addition, support groups could provide practical advice and share experiences, which can be valuable for patients navigating the complexities of their condition. The empathy from these groups could boost a patient's morale, positively affecting their mental health and motivating them to adhere to treatment plans and lifestyle changes. In essence, support from family, friends, and community networks is a cornerstone of holistic care, contributing significantly to the recovery of CVD patients.

Barriers to mental health care in cardiovascular disease management

Patients with CVDs face significant barriers to accessing mental health care, affecting their treatment and recovery. Stigma remains a significant obstacle, with many patients reluctant to seek help for mental health issues due to societal perceptions or personal beliefs [42]. Stigmas surrounding mental illness are prevalent across the Western world and can include even well-educated healthcare professionals. However, progress has been made in addressing mental illness stigma through increasing awareness, fostering open dialogue, and providing support for those affected [43]. The efforts should aim at educating the public, healthcare providers, and policymakers about the profound impact mental health has on recovery and QOL for CVD patients. Awareness campaigns should aim to clear up wrong ideas and biases about mental illness, making getting help as normal as seeking treatment for physical health issues. Furthermore, many regions, especially Africa and South East Asia (affecting over 85% of the world's population), have a big shortage of mental health resources in heart care programs [44]. These areas often have minimal funding and lack the necessary infrastructure and workforce for mental health. This leads to restricted access to specialized care. To overcome these challenges, improving telehealth, adding mental health care to basic health services, and investing more in mental health in areas that lack services are important strategies for ensuring equitable care. Additionally, there are disparities in outcomes based on race, with African-American communities facing significant obstacles. For instance, the prevalence of depression is notably higher among non-Hispanic African-Americans (9.2%) and Hispanics (8.2%) than among non-Hispanic Whites (7.9%) and non-Hispanic Asians (3.1%) [45]. Furthermore, African Americans with depression are less likely to seek treatment compared to their White counterparts [46]. Additionally, living in areas with racial/ethnic segregation has been linked to a 12% increased risk of CVDs in African-American individuals compared to White individuals [47]. These disparities place African-American and minority communities at an increased risk for both CVD complications and mental health issues. To mitigate these issues, targeted interventions such as culturally sensitive healthcare programs, community-based health outreach initiatives, and policies to reduce healthcare inequalities for underserved communities are essential. Other reasons for the limited access to mental health services among patients with CVDs are physical limitations and a lack of social support, making it difficult for these individuals to attend appointments [48,49]. To mitigate this, solutions could include providing home-based mental health services and developing community support networks to facilitate access to care for those with mobility and social challenges.

Policy and healthcare system changes to support integrated care

Policy changes and healthcare system reforms are necessary to integrate mental health care into cardiovascular disease management. Firstly, policies should include mental health services as a fundamental part of cardiovascular care, ensuring that these services are easily accessible to patients. This requires adequate funding and the development of multidisciplinary teams with cardiologists, psychiatrists, psychologists, and primary care providers. Secondly, insurance policies should be restructured to provide coverage for health care, removing financial barriers that may discourage patients from seeking the treatment they need. Thirdly, training and educating healthcare providers on how to recognize and treat mental health issues in cardiac patients is crucial. This fosters early intervention and effective management of comorbid conditions. Fourthly, there's a need for better data sharing across healthcare systems to facilitate coordinated care and follow-up. Lastly, policy changes should also address disparities in healthcare access based on race and socioeconomic status. By implementing these reforms, healthcare systems can move towards a more holistic, patient-centered model of care that addresses the interplay between mental health and cardiovascular disease management.

Future directions

The global population of older adults is rapidly growing, with projections showing an increase from 6.9% to 12% between 2000 and 2030 due to advancements in medicine and nutrition [50]. Interventions once considered underutilized in specific CVD-affected populations are gaining increasing recognition among healthcare providers. Notably, procedures such as percutaneous coronary intervention, ablation, and cardiothoracic surgeries are becoming more prevalent among older adults [51,52,53]. This trend suggests that invasive procedures on older people are gaining acceptance and will likely continue to grow in number. Additionally, regenerative medicine advancements, such as stem cell therapy and tissue engineering, offer promising possibilities for repairing and rehabilitating tissue in CVDs [54]. This innovative field is rapidly progressing. By focusing on cardiac repair through regenerative medicine, mental health challenges associated with CVDs could also be addressed. There is a growing recognition that mental health can positively influence cardiovascular health and reduce the risk of complications [25]. However, clinical medicine for patients tends to focus on the physical aspects of illness while neglecting mental health integration. Therefore, there is a need for a shift in the risk assessment paradigm towards including health and psychological factors. Furthermore, wearable technology (e.g. headbands, smartwatches, sensors embedded in clothing) has the potential to revolutionize the monitoring and early detection of CVDs [55,56]. Advanced wearables facilitate cost-effective electrocardiographic and blood pressure monitoring, aiding in the detection of cardiac arrhythmias and hypertension management [57]. This could reduce hospital visits while empowering individuals to take a role in maintaining their heart health. Furthermore, it is crucial for healthcare professionals to incorporate AI advancements into their practices. For instance, Nowakowska et al. highlighted AI's potential, showing a 62% accuracy in early depression detection among CVD patients [58]. In conclusion, the emerging trends mentioned above, coupled with a greater focus on holistic, patient-centered care, herald a more comprehensive and effective approach to treating cardiovascular diseases in the future.

## Conclusions

In conclusion, the relationship between CVDs and mental health is complex and bidirectional, where heart conditions can precipitate mental health issues, and mental health problems can, in turn, exacerbate or increase the risk of CVDs. It requires a comprehensive approach to patient care, acknowledging the need to address the cardiovascular and mental health pathologies directly and the underlying factors contributing to these conditions. Effective management strategies encompass various interventions, including pharmacological treatments, psychotherapy, lifestyle modifications, and digital health technologies to enhance patient care and support. The role of family, friends, and support groups cannot be overstated, as they provide essential emotional backing and a sense of community that significantly impacts recovery and well-being. Lastly, policy changes and healthcare system reforms play a crucial role in facilitating this integrated care model, ensuring mental health services are an integral part of cardiovascular care, improving healthcare access across diverse populations, and addressing disparities in care. By implementing these comprehensive strategies, healthcare systems can move towards a more holistic, patient-centered approach that effectively addresses the intricate interplay between the heart and mind, ultimately leading to improved outcomes for patients with CVDs and associated mental health issues.
